# Frequency of Resistance to Benzimidazoles of *Haemonchus contortus* Helminths from Dairy Sheep, Goats, Cattle and Buffaloes in Greece

**DOI:** 10.3390/pathogens9050347

**Published:** 2020-05-03

**Authors:** Konstantinos Arsenopoulos, Styliani Minoudi, Isaia Symeonidou, Alexandros Triantafyllidis, Angeliki I. Katsafadou, Daphne T. Lianou, George C. Fthenakis, Elias Papadopoulos

**Affiliations:** 1 Laboratory of Parasitology and Parasitic Diseases, School of Veterinary Medicine, Faculty of Health Sciences, Aristotle University of Thessaloniki, 54124 Thessaloniki, Greece; arsenopo@vet.auth.gr (K.A.); isaia@vet.auth.gr (I.S.); 2 Laboratory of Genetics, Development and Molecular Biology, School of Biology, Aristotle University of Thessaloniki, 54124 Thessaloniki, Greece; sminoudi@bio.auth.gr (S.M.); atriant@bio.auth.gr (A.T.); 3 Veterinary Faculty, University of Thessaly, 43100 Karditsa, Greece; agkatsaf@vet.uth.gr (A.I.K.); dlianou@vet.uth.gr (D.T.L.); gcf@vet.uth.gr (G.C.F.)

**Keywords:** allele-specific PCR, benzimidazole resistance, buffalo, cattle, goat, *Haemonchus contortus*, sheep

## Abstract

The study investigated the presence of resistance to benzimidazoles in *Haemonchus contortus* helminths from ruminant species in Greece through the detection of the Phe/Tyr polymorphism in the amino acid at position 200 of the *β*-tubulin protein. In total, 288 adult female *H. contortus* helminths collected from the abomasum of various ruminant animals in Greece were tested. Of these, 96 were collected from sheep, 96 from goats, 48 from cattle, and 48 from buffaloes. The frequencies of the homozygous and heterozygous resistant genotypes at the position 200 of the *β*-tubulin gene of helminths recovered from sheep were 96.9% and 3.1%, respectively. The frequencies of the homozygous and heterozygous resistant genotypes, respectively, were 100.0% and 0.0% in helminths from goats, 25.0% and 75.0% in helminths from cattle and 8.3% and 91.7% in helminths from buffaloes. In all parasitic populations, no homozygous susceptible genotypes were detected. The present study highlighted, for the first time, the emergence of benzimidazole-resistant *H. contortus* in goats, cattle, and buffaloes in Greece, using an allele-specific PCR. It is postulated that benzimidazole-resistant alleles were transferred from sheep or goats to cattle and buffaloes at the commonly grazing pastures in Greece.

## 1. Introduction

Gastrointestinal nematodes (GIN) lead to a significant decrease of productivity in grazing ruminants worldwide [1,2,3]. Nematode parasites, e.g., *Haemonchus* spp., *Teladorsagia circumcincta* and *Trichostrongylus* spp. lead to severe constraints in ruminant production, as the respective infections cause a reduction in milk and meat production, incur veterinary expenses, and may also lead to the death of the affected animals during severe infections [4,5,6].

Among GIN, helminths of the genus *Haemonchus* are considered (along with *Teladorsagia* spp.) to be the most pathogenic parasites of ruminants due to their blood feeding activity [7,8]. The genus *Haemonchus* includes over 10 species, within which *Haemonchus contortus* and *Haemonchus placei* are the two most frequently occurring helminths. *H. contortus* infects predominantly grazing sheep and goats, while *H. placei* has been detected mainly in large ruminants, e.g., cattle and buffaloes. However, the two species have also been recovered from other domesticated or wild ruminant populations and have been identified in mixed- or single-species infections [7,8,9,10].

Administration of antiparasitic drugs for the control of these parasites is compromised by anthelmintic resistance, which nowadays has become a significant worldwide threat [11,12,13]. Resistant helminths possess the genetic ability to counteract antiparasitic chemical action and can transmit these genes to their offspring [14].

Many reports from European countries have been published regarding anthelmintic resistance, referring to benzimidazole or levamisole resistance [15,16,17], as well as to resistance to macrocyclic lactones, including ivermectin [18,19], doramectin [20], and moxidectin [21]. Resistance to triclabendazole has also been described [22].

Nowadays, molecular evaluation of benzimidazole resistance is considered to be the most sensitive and advanced approach. More specifically, benzimidazole resistance in nematodes can be detected by molecular tests that detect a single nucleotide polymorphism (SNP) in the gene encoding isotype 1 of the *β*-tubulin that resides at codons 200, 167, and 198. In the case of *H. contortus*, a point mutation (i.e., TTC to TAC) at codon 200 [23] and 167 [24] leads to a phenylalanine to tyrosine substitution, which then can lead to benzimidazole resistance. Last but not least, another point mutation (i.e., GCA to GAA) at codon 198 leads to a substitution of glutamate into alanine, developing benzimidazole resistance [25]. Allele-specific PCR is a well-known method for the detection of point mutations, small deletions and insertions, polymorphisms, and other sequence variations in parasite DNA [26].

Most GIN genetic analyses regarding benzimidazole resistance have been carried out in *H. contortus* strains from small ruminants [27,28,29,30]. In Greece, the first report of benzimidazole resistance against *H. contortus* from sheep was reported by Gallidis et al. [16]. Those authors examined 40 adult *H. contortus* using molecular techniques, to detect point mutation at codon 200 in the gene encoding isotype 1 of the *β*-tubulin. They found 100% homozygous benzimidazole-resistant strains. Studies on the resistance of *Haemonchus* from other ruminant species in Greece have not been published. In general, benzimidazole resistance is increasing in large ruminants, which would pose a challenge to the respective industries [31,32]. Indeed, reports about benzimidazole-resistant *Haemonchus* spp. helminths from cattle [33,34,35,36] or buffaloes [17,33] have been published.

The study investigated the presence of resistance to benzimidazoles in *H. contortus* helminths from ruminant species in Greece through the detection of the Phe/Tyr polymorphism in the amino acid at position 200 of the *β*-tubulin protein.

## 2. Results

Of the 288 *H. contortus* helminths evaluated during the study, 205 (71.2%) were found to be homozygous benzimidazole-resistant, and 83 (28.8%) were found to be heterozygous benzimidazole-resistant (as found by targeting the position 200 of the *β*-tubulin protein of the helminths) (Table 1). There was a clear difference in the frequency of susceptible/susceptible, resistant/susceptible, and resistant/resistant helminths recovered among the four animal species from which the helminths were collected (*P* < 0.001). Differences in frequencies between helminths recovered from sheep or goats and cattle or buffaloes were always significant (*P* < 0.001), whilst differences in frequencies between helminths recovered from sheep and goats and between cattle and buffaloes were not (*P* > 0.052) (Table 1).

**Figure 1:**
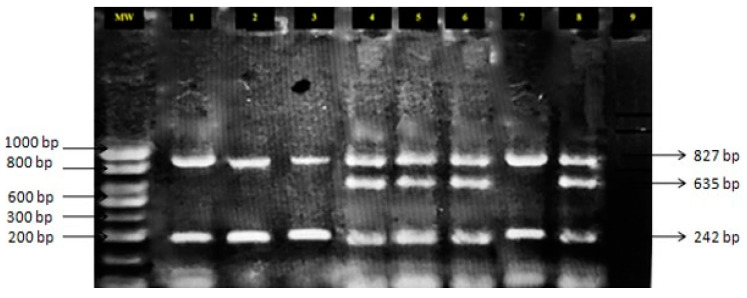
Allele-specific PCR profile of *β*-tubulin isotype 1 gene in field isolates of *Haemonchus contortus* from cattle (MW: molecular weight markers, 1: homozygous resistant positive control, 2–7: *H. contortus* genotyped, 8: heterozygous resistant positive control, 9: negative control).

Differences in the frequency of the susceptible/susceptible, resistant/susceptible, and resistant/resistant helminths recovered among the regions of the country were statistically significant for helminths recovered from cattle or buffaloes (*P* < 0.001), but not for helminths recovered from sheep or goats (*P* > 0.78) (Supplementary Material S1–S4). It became evident that in the region of Thessaly, only resistant/resistant helminths were recovered from both cattle and buffaloes. The allele-specific PCR profile of the helminths is presented in Figure 1.

## 3. Discussion

Benzimidazoles are anthelmintics, widely used worldwide against parasites of domestic ruminants and humans [11]. These drugs have a broad-spectrum anthelmintic activity, are low cost, and have a short withdrawal period [37], which all support their widespread use for nematode control. Consequently, resistance and reduced efficacy against GIN, including *H. contortus*, have developed [38]. In Greece, Gallidis et al. [16] have evaluated *H. contortus* helminths from sheep and detected SNP in the amino acid at position 200 of the gene encoding isotype-1 of *β*-tubulin. They reported only the occasional presence of homozygous resistant alleles, i.e., Tyr/Tyr. In contrast, in the present study, homozygous resistant alleles were detected in most *H. contortus* helminths (96.9% of 96 helminths collected from sources across the country), whilst the homozygous susceptible alleles (TTC/TTC) were not found.

Despite the importance of goats in Greece and in Southern European countries in general, benzimidazole resistance in *H. contortus* helminths from these animals had not been evaluated before. In a previous study [39], benzimidazole resistance in *T. circumcincta* from goats has been reported, with a frequency of 20% among the individual helminths evaluated. In the present study, all *H. contortus* helminths from goats were found to be with homozygous resistant alleles (i.e., 100%), which is much higher than the one previously reported for *T. circumcincta* [39].

The present findings provide evidence of widespread benzimidazole resistance of *H. contortus* in sheep and goat farms in Greece. The collection of samples from farms around the country lends strength to the study and indicates the widespread presence of benzimidazole resistance. To the best of our knowledge, the present findings are the first ones in Europe that describe such widespread resistance of *H. contortus* to benzimidazoles.

The findings point out to a sharp increase in the frequency of resistance since the previous relevant works. This is mainly the consequence of increased and frequent use of anthelmintics in the years following the previous studies. In a previous field investigation, it was found that benzimidazoles were the principal class of anthelmintics used in 91% of small ruminant farms in Greece, with 2.5 (sheep) to 2.6 (goats) mean administrations annually. Macrocyclic lactones, the second most frequently used class, was administered by 51% of farmers [40]. Less likely, the findings can reflect improved sensitivity of the technique employed in the current work or differences between parasitic species evaluated in the present or the previous works. It is noteworthy that in Greece sheep and goats routinely graze in common pastures, and consequently, they share the same GIN helminths [41]. This can favor dissemination of benzimidazole-resistant *H. contortus* between the two animal species [42] and would contribute to a wider expansion of resistant helminths.

Although many studies have been performed about benzimidazole resistance in *H. contortus* helminths from sheep or goats, there are only few references regarding that resistance in *H. contortus* helminths recovered from cattle or buffaloes. The detection of the specific SNP, which leads to tyrosine (Tyr) appearance at the position 200 of the gene encoding isotype 1 of *β*-tubulin in helminths from large ruminants, revealed some presence of resistance also in these parasites: 25% homozygous resistant (Tyr/Tyr) alleles in helminths from cattle and 8.3% in helminths from buffaloes. These findings indicate the emergence of the TAC resistance, conferring mutations in *H. contortus* among populations of the parasite in these two animal species.

Grazing of these animals in common pastures with sheep or goats can plausibly explain a potential dissemination of benzimidazole-resistant alleles from sheep and goats to cattle and buffaloes and lends support to a hypothesis regarding a possible role of co-grazing in the spread of resistant alleles. Co-grazing of small ruminants with cattle and buffaloes has been documented in Greece [43] as the result of incorrect management of pasturelands. In the 1990s, no resistance to benzimidazoles was detected in helminths recovered from cattle in Greece [44]. Anecdotal evidence, including the clinical experience of the senior authors (EP, GCF), indicates that cattle and buffaloes in Greece are not routinely treated with anthelmintics. Hence, one can postulate that the recovery of gastrointestinal helminths with some resistance to benzimidazoles from these animals can be the result of co-grazing rather than a direct effect of the administration of anthelmintics. However, the development of resistance was slower than in sheep or goats given the lack of anthelmintic administration. Further support to this hypothesis can be provided by the recovery of resistant/resistant helminths from cattle and buffaloes in the region of Thessaly, as that part of the country has a large population of sheep and goats (12% of small ruminant numbers and farms of the country [45,46]). In an ongoing study, 83.5% of these animals were found to graze daily [47]. Nevertheless, there may also be a benefit in the situation: the presence in refugia of heterozygous alleles among helminths from cattle or buffaloes can be used for a ‘dilution’ of the homozygous resistant strains in these commonly grazed fields, and consequently, the increase of susceptible alleles in small ruminant *H. contortus*. This approach can provide an option for the decrease of resistance to benzimidazoles of *H. contortus* in sheep and goat farms. Phenotypically, presence of anthelmintic resistance would be considered in cases of inefficacy of the veterinary drugs administered for control of the parasitic infections. Often, this would be overcome by increasing the dose rate of the antiparasitics. In such cases, increased withdrawal periods after drug administration would be necessary, but might not be maintained always, which in turn would lead to increased drug residues in products for human consumption. This can be a one-health issue of gastrointestinal parasitism of small ruminants.

The mechanisms of resistance to benzimidazoles has been studied in sheep and goat helminths, including *H. contortus*, and there is strong evidence that three different single amino acid substitutions in the isotype 1 of the *β*-tubulin are responsible. Resistance can arise in cases of a SNP in the gene encoding isotype 1 of the *β*-tubulin, at codons 200, 167, or 198. In the case of *H. contortus*, a point mutation (i.e., TTC to TAC) at codon 200 [23] or 167 [24] leads to a phenylalanine (Phe) to tyrosine (Tyr) substitution, which in turn leads to benzimidazole resistance. A point mutation (i.e., GCA to GAA) at codon 198 causing substitution of glutamate into alanine can also lead to development of resistance [25]. It is noteworthy that, after using appropriate proteomics methodologies, Umair et al. [48] reported that amino acid sequences (including that of *β*-tubulin) in *H. contortus* and *T. circumcincta* showed similarity with each other. After performing a specific protein sequence alignment between *β*-tubulin of *H. contortus* and *T. circumcincta* in UniProt Knowledge base database, the similarity (99%) between the two proteins was confirmed. This further supports the validity of the techniques used in the study.

Among these mutations, the first, at codon 200, is the one occurring most frequently, causing widespread benzimidazole resistance in helminths from small ruminants [49,50,51]. The mutation at codon 167 has been detected in some countries, but always at lower frequencies than the one at codon 200 [52,53,54]. Only Redman et al. [51] the United Kingdom reported the frequency of this mutation to be higher than the one in codon 200. The mutation at codon 198 is considered even more rare and has been reported only thrice [25,29,55]. Although mutations in one of the above codons can lead to benzimidazole resistance in *H. contortus*, examination of *β*-tubulin genotypes of individual *H. contortus* helminths has suggested that combinations of two or three of these generic changes would not occur in the same *β*-tubulin isotype-1 allele. This may indicate that multiple mutations in the same allele can cause the death of the respective helminth [52,56].

The frequencies of these three mutations in *H. contortus* helminths from cattle or buffaloes have not been estimated before. Ali et al. [17] found that the most frequent mutation in *H. contortus* helminths from large ruminants was related with the position 200 at isotype-1 of *β*-tubulin and ranged from 7 to 57%, whilst the other two mutations (i.e., at codons 167 and 198) were not detected. In another study, however, the frequency (0–2.5%) of mutation at codon 167 was higher than that of (0%) at codon 200 in helminths from cattle [33]. No relevant information could be found regarding frequency of mutations in helminths from buffaloes.

In conclusion, the present study, in which an allele-specific PCR was used, highlighted for the first time the extensive presence of resistance to benzimidazoles in *H. contortus* recovered from small or large ruminants in Greece. It is postulated that possibly, these helminths spread from sheep and goats to cattle and buffaloes at common grazing pastures. Despite the high frequency of the homozygous benzimidazole-resistant alleles in *H. contortus* helminths from small ruminants, helminths from large ruminants were found to be mostly heterozygous and maintained some susceptibility to benzimidazoles. This can be viewed as a potential option for the decrease of benzimidazole-resistant *H. contortus* helminths in small ruminants through the presence in refugia and the subsequent dissemination of the susceptible alleles to sheep and goats.

## 4. Materials and Methods

### 4.1. Helminths

**Figure 2:**
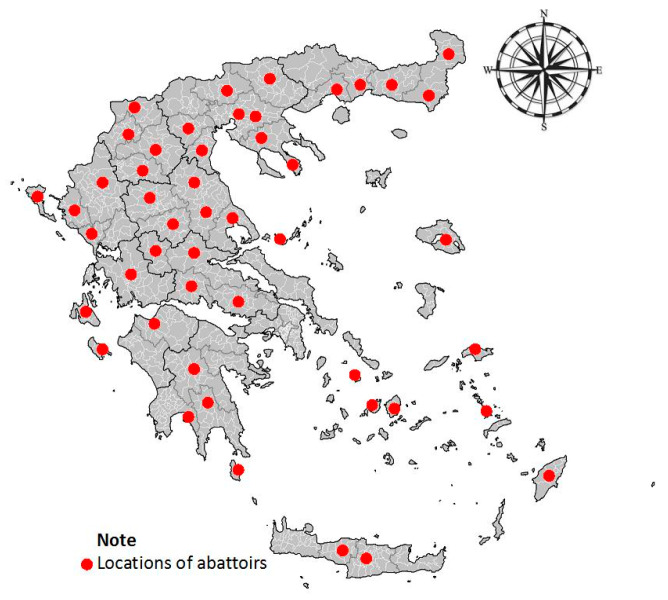
Location throughout Greece of abattoirs, where *Haemonchus contortus* helminths were collected from sheep, goats, cattle, or buffaloes, for evaluation of their susceptibility/resistance to benzimidazoles.

*Haemonchus* spp. helminths were collected directly from the abomasum of ruminants (sheep, goats, cattle, and buffaloes), immediately after slaughter of the animals at various abattoirs throughout Greece (Figure 2). These were selected throughout Greece on convenience basis, i.e., if collection of material from slaughtered animals was permitted. In each abattoir, carcasses for material collection were selected at random. In total, 171 abomasa from respective animals (from 146 farms) were taken for parasite collection. Of these, 55 were from sheep (from 53 farms), 63 from goats (from 55 farms), 28 from cattle (from 25 farms), and 24 from buffaloes (from 13 farms). Details are shown in Supplementary Material S1–S4.

After slaughter, the abomasum of each animal was removed and dissected. From each abomasum, 1 to 3 *Haemonchus* spp. helminths were picked at random for evaluation. They were collected into vials with ethanol 99%, which were transferred to the laboratory within 18 h and stored at 4 °C. Finally, 288 female *Haemonchus* spp. helminths (96 from sheep, 96 from goats, 48 from cattle, and 48 from buffaloes) were collected for evaluation. Details are shown in Supplementary Material S1–S4.

Detailed identification of the *Haemonchus* helminths recovered was performed on the basis of spicule length and morphology and the characteristics of the pattern of the longitudinal ridges (synlophe) on the external cuticular surface [7] of the parasite. All the helminths evaluated during the study were fully identified as *H. contortus*.

### 4.2. DNA Extraction—Allele Specific PCR

The head of each individual helminth was dissected from the rest of the body at the cervical papillae, excluding the parasite’s uterus and eggs (in order to avoid any genetic material shared by male parasites), and was taken for DNA extraction. Whole genomic DNA was extracted using the protocol of Hillis et al. [57], which is based on the chemical compound cetyl-trimethyl-ammonium bromide. After DNA extraction, tubes containing DNA were stored at −20 °C. The quality of the extracted DNA was evaluated by electrophoresis on 1% agarose gels stained with ethidium bromide and visualized on a UV transilluminator.

For targeting the position 200 of the *β*-tubulin protein of the helminths, a previously described allele-specific multiplex PCR was employed [58,59]. Four different primers (P1, P4, P2S, P3R) (Eurofins Genomics Germany GmbH, Ebersberg, Germany) were used in the same reaction mix (Table 2). PCR amplification was performed in 10 μL reaction mixtures, containing 1 μL DNA, 5 μL KARA Taq ReadyMix PCR kit (content: 1 unit per 50 μL reaction KAPA Taq DNA polymerase, 0.2 mM of each deoxynucleoside triphosphate [dNTP], 1.5 mM magnesium chloride and stabilizers), 1.2 μL double distilled water and the primers (Table 2), in a Takara PCR thermal cycler Dice (Takara BIO INC., Shiga, Japan). The temperature cycling protocol (Table 2) was repeated 40 times. Each PCR reaction was initiated with a 5 min denaturation at 94 °C and terminated with a 5 min extension at 72 °C. PCR amplicons were analyzed by electrophoresis on 1.5% agarose gels stained with ethidium bromide and visualized on a UV transilluminator.

### 4.3. Data Management and Analysis

The frequency of susceptible/susceptible, resistant/susceptible, and resistant/resistant helminths recovered from the animals was compared among the four animal species in a 4 × 3 Chi-square test evaluation. The difference between the regions of the country in the frequency of susceptible/susceptible, resistant/susceptible, and resistant/resistant helminths recovered from the animals was compared among the regions in a 3 × i Chi-square test evaluation (i = 13 for sheep and goats, 7 for cattle, and 2 for buffaloes). The Fisher exact test was used to compare the frequencies between each of two animal species. Statistical significance was defined at *P* < 0.05.

## Figures and Tables

**Table 1:** Frequency of susceptible/susceptible (S/S), resistant/susceptible (R/S), and resistant/resistant (R/R) *Haemonhus contortus* helminths from four ruminant species in Greece (as found by targeting the position 200 of the *β*-tubulin protein of the helminths).

Species	Susceptibility/Resistance Status (%)			
	S/S	R/S	R/R	Total
Sheep	0 (0.0%)	3 (3.1%)	93 (96.9%)	96 (100.0%)
Goats	0 (0.0%)	0 (0.0%)	96 (100.0%)	96 (100.0%
Cattle	0 (0.0%)	36 (75.0%)	12 (25.0%)	48 (100.0%)
Buffaloes	0 (0.0%)	44 (91.7%)	4 (8.3%)	48 (100.0%)
Total	0 (0.0%)	83 (28.8%)	205 (71.2%)	288 (100.0%)

**Table 2:** Primers used and work conditions undertaken for an allele-specific PCR for targeting the position 200 of the *β*-tubulin protein of the *Haemonchus contortus* helminths.

**Primer**	**Sequence**		**Quantity in Reaction Mixture**
P1	Fw: 5′-GTCCCACGTGCTGTTCTTGT -3′		0.4 μL
P2S	Rv: 5′-TACAGAGCTTCATTATCGATGCAGA -3′		1.0 μL
P3R	Fw: 5′-TTGGTAGAAAACACCGATGAAACATA-3′		1.0 μL
P4	Rv: 5′-GATCAGCATTCAGCTGTCCA-3′		0.4 μL
**Working Conditions**			
**Target**	**Primer Pair**	**Product Size (bp)**	**Cycling Protocol**
Presence of parasite	P1/P4	827	Denaturation: 94 °C for 30 s; Annealing: 57 °C for 30 s; Extension: at 72 °C for 45 s
Susceptible alleles	P1/P2S	635	
Resistant alleles	P3R/P4	242	

## Supplementary Materials

The following are available online at https://www.mdpi.com/2076-0817/9/5/347/s1, Table S1: Details of geographical origin of *Haemonchus contortus* helminths from sheep in Greece and results of their susceptibility/resistance to benzimidazoles, Table S2: Details of geographical origin of *Haemonchus contortus* helminths from goats in Greece and results of their susceptibility/resistance to benzimidazoles, Table S3: Details of geographical origin of *Haemonchus contortus* helminths from cattle in Greece and results of their susceptibility/resistance to benzimidazoles, Table S4: Details of geographical origin of *Haemonchus contortus* helminths from buffaloes in Greece and results of their susceptibility/resistance to benzimidazoles.
