# Supplementary material for: Frequency of Resistance to Benzimidazoles of Haemonchus contortus Helminths from Dairy Sheep, Goats, Cattle and Buffaloes in Greece
Source: Pathogens. 2020 May 3;9(5):347. doi: 10.3390/pathogens9050347 (PMC7280990; doi:10.3390/pathogens9050347)
Supplement: Supplementary file 1 [file pathogens-09-00347-s001.pdf]

*Supplementary material*

*Article*

# Frequency of Resistance to Benzimidazoles of *Haemonchus contortus* Helminths from Dairy Sheep, Goats, Cattle and Buffaloes in Greece

**Konstantinos Arsenopoulos, Styliani Minoudi, Isaia Symeonidou, Alexandros Triantafyllidis, Angeliki I. Katsafadou, Daphne T. Lianou, George C. Fthenakis and Elias Papadopoulos**

**Table S1.** Details of geographical origin of *Haemonchus contortus* helminths from sheep in Greece and results of their susceptibility / resistance to benzimidazoles.

| Region of the country | Local authority | Flocks (n) | Abomasa (n) | *H. contortus* helminths (n) | Susceptibility / Resistance status of helminths (n) | | |
|---|---|---|---|---|---|---|---|
| | | | | | S / S | R / S | R / R |
| East Macedonia and Thrace | Kavala | 2 | 2 | 4 | 0 | 0 | 4 |
| | Rodopi | 1 | 1 | 2 | 0 | 0 | 2 |
| | Xanthi | 1 | 1 | 2 | 0 | 0 | 2 |
| Central Macedonia | Kilkis | 1 | 1 | 1 | 0 | 0 | 1 |
| | Pieria | 2 | 2 | 4 | 0 | 0 | 4 |
| | Serres | 2 | 2 | 4 | 0 | 0 | 4 |
| | Thessaloniki | 7 | 7 | 12 | 0 | 0 | 12 |
| West Macedonia | Grevena | 2 | 2 | 4 | 0 | 1 | 3 |
| | Kastoria | 1 | 1 | 2 | 0 | 0 | 2 |
| Epirus | Ioannina | 1 | 1 | 2 | 0 | 0 | 2 |
| Thessaly | Karditsa | 3 | 3 | 6 | 0 | 0 | 6 |
| | Larissa | 6 | 6 | 12 | 0 | 0 | 12 |
| | Magnisia | 1 | 1 | 2 | 0 | 0 | 2 |
| | Sporades | 2 | 2 | 3 | 0 | 1 | 2 |
| | Trikala | 1 | 1 | 2 | 0 | 0 | 2 |
| Ionian islands | Corfu | 1 | 1 | 1 | 0 | 0 | 1 |
| | Kefallonia | 1 | 1 | 1 | 0 | 0 | 1 |
| | Zakynthos | 1 | 1 | 1 | 0 | 0 | 1 |
| West Greece | Achaia | 1 | 1 | 1 | 0 | 0 | 1 |
| Continental Greece | Evritania | 1 | 1 | 1 | 0 | 0 | 1 |
| | Fthiotida | 1 | 1 | 2 | 0 | 0 | 2 |

| | | | | | | | |
|---|---|---|---|---|---|---|---|
| | Viotia | 1 | 1 | 2 | 0 | 0 | 2 |
| Attica | Attica islands | 3 | 3 | 3 | 0 | 0 | 3 |
| Peloponnese | Arkadia | 1 | 1 | 2 | 0 | 0 | 2 |
| | Messinia | 1 | 1 | 2 | 0 | 0 | 2 |
| North Aegean | Lesvos | 1 | 1 | 2 | 0 | 0 | 2 |
| | Samos | 1 | 1 | 2 | 0 | 0 | 2 |
| South Aegean | Kalymnos | 2 | 3 | 5 | 0 | 1 | 4 |
| | Paros | 1 | 1 | 2 | 0 | 0 | 2 |
| | Rhodes | 1 | 2 | 4 | 0 | 0 | 4 |
| Crete | Iraklion | 1 | 1 | 1 | 0 | 0 | 1 |
| | Rethymnon | 1 | 1 | 2 | 0 | 0 | 2 |
| | **TOTAL** | **53** | **55** | **96** | **0** | **3** | **93** |

**Table S2.** Details of geographical origin of *Haemonchus contortus* helminths from goats in Greece and results of their susceptibility / resistance to benzimidazoles.

| Region of the country | Local authority | Flocks (n) | Abomasa (n) | *H. contortus* helminths (n) | Susceptibility / Resistance status of helminths (n) | | |
|---|---|---|---|---|---|---|---|
| | | | | | S / S | R / S | R / R |
| East Macedonia and Thrace | Evros | 1 | 2 | 2 | 0 | 0 | 2 |
| | Kavala | 1 | 1 | 2 | 0 | 0 | 2 |
| | Rodopi | 2 | 2 | 2 | 0 | 0 | 2 |
| | Xanthi | 1 | 1 | 2 | 0 | 0 | 2 |
| Central Macedonia | Chalkidiki | 2 | 3 | 4 | 0 | 0 | 4 |
| | Kilkis | 1 | 2 | 2 | 0 | 0 | 2 |
| | Pieria | 1 | 1 | 2 | 0 | 0 | 2 |
| | Serres | 2 | 2 | 4 | 0 | 0 | 4 |
| | Thessaloniki | 2 | 2 | 4 | 0 | 0 | 4 |
| West Macedonia | Florina | 1 | 1 | 2 | 0 | 0 | 2 |
| | Grevena | 1 | 1 | 3 | 0 | 0 | 3 |
| | Kastoria | 1 | 1 | 2 | 0 | 0 | 2 |
| Epirus | Ioannina | 1 | 1 | 1 | 0 | 0 | 1 |
| | Preveza | 1 | 1 | 2 | 0 | 0 | 2 |
| | Thesprotia | 1 | 1 | 1 | 0 | 0 | 1 |
| Thessaly | Karditsa | 2 | 2 | 4 | 0 | 0 | 4 |
| | Larissa | 2 | 2 | 4 | 0 | 0 | 4 |
| | Magnisia | 2 | 2 | 4 | 0 | 0 | 4 |
| | Sporades | 3 | 3 | 3 | 0 | 0 | 3 |
| | Trikala | 2 | 4 | 6 | 0 | 0 | 6 |
| Ionian islands | Corfu | 1 | 1 | 1 | 0 | 0 | 1 |
| | Kefallonia | 1 | 1 | 1 | 0 | 0 | 1 |
| | Zakynthos | 1 | 1 | 1 | 0 | 0 | 1 |
| West Greece | Achaia | 1 | 1 | 1 | 0 | 0 | 1 |
| | Aitoloakarnania | 1 | 2 | 2 | 0 | 0 | 2 |
| Continental Greece | Evritania | 1 | 1 | 2 | 0 | 0 | 2 |
| | Fokida | 1 | 1 | 2 | 0 | 0 | 2 |
| | Fthiotida | 1 | 1 | 1 | 0 | 0 | 1 |
| | Viotia | 1 | 2 | 2 | 0 | 0 | 2 |
| Attica | Attica islands | 1 | 1 | 2 | 0 | 0 | 2 |
| Peloponnese | Arkadia | 1 | 1 | 2 | 0 | 0 | 2 |
| | Laconia | 1 | 1 | 2 | 0 | 0 | 2 |

| | | | | | | | |
|---|---|---|---|---|---|---|---|
| North Aegean | Lesvos | 2 | 3 | 4 | 0 | 0 | 4 |
| | Samos | 1 | 1 | 2 | 0 | 0 | 2 |
| South Aegean | Kalymnos | 3 | 3 | 5 | 0 | 0 | 5 |
| | Paros | 2 | 2 | 2 | 0 | 0 | 2 |
| | Syros | 1 | 1 | 2 | 0 | 0 | 2 |
| | Rhodes | 2 | 2 | 3 | 0 | 0 | 3 |
| Crete | Iraklion | 1 | 1 | 1 | 0 | 0 | 1 |
| | Rethymnon | 1 | 1 | 2 | 0 | 0 | 2 |
| | **TOTAL** | **55** | **63** | **96** | **0** | **0** | **96** |

**Table S3.** Details of geographical origin of *Haemonchus contortus* helminths from cattle in Greece and results of their susceptibility / resistance to benzimidazoles.

| Region of the country | Local authority | Flocks (n) | Abomasa (n) | *H. contortus* helminths (n) | Susceptibility / Resistance status of helminths (n) | | |
|---|---|---|---|---|---|---|---|
| | | | | | S / S | R / S | R / R |
| East Macedonia and Thrace | Kavala | 2 | 2 | 3 | 0 | 2 | 1 |
| | Rodopi | 3 | 3 | 6 | 0 | 4 | 2 |
| | Xanthi | 1 | 1 | 2 | 0 | 1 | 1 |
| Central Macedonia | Chalkidiki | 1 | 1 | 2 | 0 | 2 | 0 |
| | Imathia | 1 | 1 | 2 | 0 | 2 | 0 |
| | Serres | 2 | 2 | 4 | 0 | 4 | 0 |
| | Thessaloniki | 5 | 5 | 8 | 0 | 8 | 0 |
| West Macedonia | Kozani | 1 | 1 | 1 | 0 | 1 | 0 |
| Epirus | Ioannina | 1 | 1 | 2 | 0 | 2 | 0 |
| | Preveza | 1 | 1 | 2 | 0 | 2 | 0 |
| Thessaly | Karditsa | 1 | 2 | 2 | 0 | 0 | 2 |
| | Larissa | 2 | 2 | 2 | 0 | 0 | 2 |
| | Magnisia | 1 | 1 | 2 | 0 | 0 | 2 |
| | Trikala | 1 | 1 | 2 | 0 | 0 | 2 |
| North Aegean | Lesvos | 1 | 1 | 2 | 0 | 2 | 0 |
| South Aegean | Naxos | 1 | 3 | 6 | 0 | 6 | 0 |
| | **TOTAL** | **25** | **28** | **48** | **0** | **36** | **12** |

**Table S4.** Details of geographical origin of *Haemonchus contortus* helminths from buffaloes in Greece and results of their susceptibility / resistance to benzimidazoles.

| Region of the country | Local authority | Flocks (n) | Abomasa (n) | *H. contortus* helminths (n) | Susceptibility / Resistance status of helminths (n) | | |
|---|---|---|---|---|---|---|---|
| | | | | | S / S | R / S | R / R |
| Central Macedonia | Pieria | 2 | 4 | 8 | 0 | 8 | 0 |
| | Serres | 6 | 11 | 22 | 0 | 22 | 0 |
| | Thessaloniki | 4 | 7 | 14 | 0 | 14 | 0 |
| Thessaly | Trikala | 1 | 2 | 4 | 0 | 0 | 4 |
| | **TOTAL** | **13** | **24** | **48** | **0** | **32** | **4** |

© 2020 by the authors. Submitted for possible open access publication under the terms and conditions of the Creative Commons Attribution (CC BY) license (http://creativecommons.org/licenses/by/4.0/).
